# Coupling relationships between yield traits of *Ipomoea aquatica* Forssk. and biogas slurry purification under varied nutrient supplementation regimes

**DOI:** 10.3389/fbioe.2026.1927455

**Published:** 2026-10-01

**Authors:** Yuhuan Wu, Lanmei Liang, Linyu Xie, Xujing Guo, Wenguo Wang, Liangwei Deng, Lan Wang, Hongwei Zhang, Ting He, Peike Wu, Yunhong Zhang

**Affiliations:** 1 College of Resources and Environment, Chengdu University of Information Technology, Chengdu, China; 2 Biogas Institute of Ministry of Agriculture and Rural Affairs, Chinese Academy of Agricultural Sciences, Chengdu, China; 3 Key Laboratory of Development and Application of Rural Renewable Energy, Ministry of Agriculture and Rural Affairs, Chengdu, China

**Keywords:** ammonia nitrogen, biogas slurry purification, COD, hydroponic *Ipomoea aquatica* Forssk., nutrient regulation, total phosphorus

## Abstract

Biogas slurry from intensive pig farms contains high concentrations of ammonium nitrogen (AN), total phosphorus (TP), and chemical oxygen demand (COD). However, its direct application in hydroponics is constrained by ammonium toxicity, nutrient imbalance, and the lack of rapid load-monitoring tools. In this study, a hydroponic system with *I. aquatica* Forssk. (*Ipomoea aquatica*) was established to simultaneously purify biogas slurry and increase vegetable production. Regression models for rapid estimation of AN and total nitrogen (TN) concentrations were developed using electrical conductivity (EC) of slurry samples from three representative Chinese regions (Northeast, Jiangsu, and Sichuan). The gradient tolerance test determined that 100 mg/L of AN is the optimal concentration for promoting vegetable yield (889 kg/mu, 1 mu = 1/15 ha), while 200 mg/L AN is the critical toxicity threshold. Factorial nutrient supplementation experiments showed that full supplementation with macro-, secondary, and micronutrients (Treatment T4) maximized plant growth, chlorophyll content, and quality indices. The system achieved >90% COD removal across all treatments, while TP removal efficiency was highly dependent on the interaction between AN load and micronutrient supply, which was antagonistic under low AN but synergistic under high AN. Economic assessment revealed an annual net income of approximately CNY 45133 per mu. The EC model is applicable for initial slurry assessment but not suitable for real-time monitoring after exogenous nutrient addition. This study provides a practical, low-cost integrated strategy for biogas slurry resource utilization and vegetable production, and offers an effective hydroponic *I. aquatica* system for small- and medium-scale pig farms.

## 1 Introduction

Biogas slurry is a byproduct of anaerobic digestion in intensive pig farming, containing high concentrations of ammonium nitrogen (AN), total phosphorus (TP), and chemical oxygen demand (COD) (Ruan et al., 2015; He et al., 2017; Tambone et al., 2017). Traditionally viewed solely as a waste liquid requiring disposal, it is increasingly recognized in modern agricultural science as a nutrient-rich bio-product and a valuable liquid fertilizer that supports the development of circular agricultural systems (Wang et al., 2019; Shafaghat et al., 2025). Nevertheless, improper management, whether via direct discharge or large-scale land application, poses a range of environmental risks, such as soil salinization, leaching of nitrogen and phosphorus into groundwater, and eutrophication of surface water bodies (Martín-Queller et al., 2010; Rivas Lucero et al., 2018). With the continued maturation of anaerobic digestion technologies, the efficient treatment and resource-oriented utilization of biogas slurry have become critical priorities in livestock pollution control (Chen et al., 2020).

Hydroponic soilless culture systems enable precise control over water and nutrient delivery, while keeping plant roots fully immersed in the nutrient solution for direct uptake of nitrogen and phosphorus (Kumar et al., 2025; Kurniawan et al., 2026). Cultivating *I. aquatica* Forssk. (*Ipomoea aquatica*) in biogas slurry-based nutrient solutions can establish a synergistic “cultivation-purification” system. In this system, *I. aquatica* roots actively assimilate AN and phosphorus, while rhizosphere microorganisms simultaneously degrade organic pollutants, achieving *in situ* slurry purification and reducing pollutant discharge risks (Liang et al., 2023). Concurrently, nutrients in the slurry are recycled, partially replacing chemical fertilizers and lowering production costs (Silkina et al., 2019; Wang et al., 2019).

In recent years, many studies have explored various strategies for integrating biogas slurry into hydroponic systems. Mahrous et al. (Mahrous et al., 2026) utilized nitrification pretreatment for the conversion of NH_4_^+^ to NO_3_^−^, a strategy that effectively averted ammonium toxicity, supported vigorous crop development, and maintained negligible human health risks. However, this approach requires dedicated nitrification reactors and aeration systems, which substantially raise operational complexity, energy consumption, and infrastructure costs, thereby limiting its applicability in small- and medium-scale farms. Direct dilution with exogenous mineral nutrient supplementation is an alternative strategy that bypasses the nitrification step, reducing both investment and operational complexity, and is particularly suited for decentralized pig farms in China. While particularly suited for decentralized pig farms in China, this approach requires precise control of initial AN levels and access to rapid monitoring to avert ammonium toxicity, while batch-to-batch variations in slurry composition often destabilizes treatment stability. These two technological pathways are complementary: nitrification-based systems are better suited for large-scale, well-managed operations, whereas the direct dilution approach provides a more economically feasibility for resource-limited decentralized farming operations.

Despite these advantages, the direct application of raw biogas slurry in hydroponics faces three major bottlenecks that severely constrain pollutant removal efficiency. Firstly, raw slurry typically contains AN at 300–2,000 mg/L. Such elevated levels disrupt pH homeostasis, suppress K^+^ uptake, and impair photosynthesis, eventually causing root rot and plant death, which removes the purification carrier (Fang et al., 2021; Xiao et al., 2022). Secondly, dilution (necessary to mitigate ammonium toxicity) reduces micronutrient concentrations below crop critical thresholds, thereby impairing photosynthesis, biomass, and root development, which collectively reduce nitrogen and phosphorus removal capacity (Zulfiqar et al., 2024). Thirdly, substantial regional and batch-wise variations in AN and total nitrogen (TN) concentrations, coupled with the lack of rapid on-site detection methods, hinder precise regulation of pollutant loading, potentially inducing phytotoxicity or inadequate purification performance (Sakuma et al., 2024; Mahrous et al., 2026). To ensure long-term system stability, rapid quantification via physicochemical parameters, coupled with dynamic load matching to plant uptake capacity, is essential (Qiu et al., 2023; Mahrous et al., 2026). Furthermore, establishing a safe AN loading threshold and developing precise nutrient supplementation protocols are essential for the simultaneous optimization of vegetable yield and contaminant removal performance.

The core objective of this study is to develop a biogas slurry-adapted hydroponic system guided by the principle of “cultivating vegetables with water and purifying water with vegetables”, addressing the three technical challenges of ammonium toxicity, nutrient imbalance, and rapid concentration monitoring. The specific aims are: (1) to develop EC-based regression models for rapid quantification of AN and TN using biogas slurry samples collected from Northeast China, Jiangsu, and Sichuan provinces, to support on-site pollutant loading monitoring; (2) to determine the optimal AN concentration for unimpaired growth of *I. aquatica* and the critical ammonium toxicity threshold through gradient AN treatments; (3) to elucidate the regulatory mechanisms of macronutrient, secondary nutrient, and micronutrient supplementation on the physiological activity and pollutant sequestration capacity of *I. aquatica* via factorial experiments; (4) to quantify the sequestration and removal efficiencies of AN, COD, and TP under varying ammonium loadings and nutrient combinations, thereby elucidating the coupled purification mechanisms; and (5) to conduct an economic accounting to evaluate the input–output benefits of this eco-purification technology. The findings are expected to provide theoretical guidance and operational protocols for the low-cost ecological elimination of nitrogen, phosphorus, and organic pollutants in biogas slurry from large-scale pig farms.

## 2 Materials and methods

### 2.1 Test materials

#### 2.1.1 Biogas slurry collection

To cover the pollution characteristics of typical large-scale pig farms in China, biogas slurry samples were collected in Northeast China (9 farms), Jiangsu Province (7 farms), and Sichuan Province (7 farms). All farms had fermentation cycles ≥30 days, ensuring complete organic matter decomposition and sufficient nutrient release. Samples were stored at 4 °C for subsequent analysis and model construction.

Slurry for the hydroponic experiments was obtained from a pig farm in Jianyang, Sichuan Province. Basic physicochemical parameters were measured upon arrival, and the slurry was stored at room temperature. Owing to differences in batch and storage time, the slurry properties for the AN tolerance test and the nutrient supplementation experiment differed slightly.

#### 2.1.2 Hydroponic plant material

Seedlings of the Willow-leaf cultivar of *I. aquatica* were purchased from a local vegetable seed market. This species was chosen because its root system exhibits high nitrogen and phosphorus uptake capacity-three times that of lettuce and four to six times that of two other Chinese cabbage varieties (Trang et al., 2010).

### 2.2 Experimental design

#### 2.2.1 Establishment of EC-Based regression models

Using EC as the independent variable and AN and TN as dependent variables, univariate linear regressions were performed on samples from the three regions, with significance set at p < 0.05. Local Sichuan slurry samples were used for model validation and coefficient correction by comparing measured and predicted concentrations; least-squares optimization improved the estimation accuracy for local conditions.

#### 2.2.2 Hydroponic purification system experiments

Planting Protocol: Seeds were sown in a substrate consisting of soil and commercial matrix (2:1, v/v). Seedlings at the 6–8 cm height stage with four fully expanded true leaves were transplanted. Detailed information on the cultivation of *I. aquatica* is provided in Supplementary Text S2. The ammonia nitrogen tolerance test was conducted in an illuminated incubator, while the exogenous nutrient supplementation experiment was carried out in an experimental field. Detailed experimental conditions and environmental parameters are provided in Text S2.

##### 2.2.2.1 Ammonium nitrogen gradient tolerance test

Raw slurry was diluted to six nominal AN concentrations: 50, 100, 150, 200, 300, and 400 mg/L. The AN concentration was detecting every 7 days, and fresh raw slurry was supplemented to bring the level back to the initial load. Each treatment was replicated three times.

Based on the tolerance results, four long-term purification treatments were set: T1 (constant 100 mg/L), T2 (constant 150 mg/L), T3 (100 mg/L initially, increased to 150 mg/L after the first harvest), and T4 (1/2-strength Yamazaki nutrient solution as a control without slurry pollutants). Plant growth, yield, and photosynthetic parameters were periodically measured. Each treatment was performed in triplicate, and the AN concentration was restored to the initial value on a weekly basis.

##### 2.2.2.2 Factorial nutrient supplementation experiment

To compensate for micronutrient losses from dilution, the biogas slurry was diluted to a base solution with an AN level of 100 mg/L. Nutrients were supplemented via two strategies: (1) biogas slurry was added every 7 days based on initial AN to replenish AN; (2) mineral elements were added only at the beginning of the experiment, following a 1/2-strength Yamazaki nutrient solution, to supplement all nutrients except nitrogen that were deficient in the biogas slurry.

The experiment included one nutrient solution control group, one biogas-slurry control group without nutrient addition, and three biogas slurry treatment groups with nutrient addition. The specific treatments were as follows: the control groups were 1/2-strength Yamazaki nutrient solution (CK) and the biogas slurry without nutrient addition group (T1); the treatment groups were the group with macronutrients added based on the elemental concentrations of the 1/2-strength Yamazaki nutrient solution (T2), the group with macronutrients and secondary nutrients added (T3), and the group with macronutrients, secondary nutrients, and micronutrients added (T4). The specific treatment groups are detailed in Table 1. Each treatment was performed in triplicate. Text S3 provides detailed information on the preparation protocol of the hydroponic solution and the chemical reagents used.

**TABLE 1:** Treatment groups and the corresponding addition concentrations of each element.

Group	Category of added elements	Specific elements	Final concentrations of corresponding elements (mg/L)
CK	—	P, K, Ca, Mg, S, Fe, Mn, B, Zn, Mo, Cu	K 156.4; P 20.62; Ca 40; Mg 24; S 32; Fe 4.56; Zn 0.05; Cu 0.02; Mn 0.52; B 0.50; Mo 0.01
T1	—	—	—
T2	Macronutrients	P, K	The concentrations of the supplemented elements were referenced to those in the CK group
T3	Macronutrients, secondary nutrients	P, K, Ca, Mg, S	
T4	Macronutrients, secondary nutrients, and micronutrients	P, K, Ca, Mg, S, Fe, Mn, B, Zn, Mo, Cu	

Due to variations in the basic physicochemical properties of the biogas slurry, the concentrations of individual elements in the diluted slurry for Group T1 differed.

##### 2.2.2.3 Pollutant removal tests

Four nutrient addition regimes (T1: no addition; T2: macronutrients; T3: macronutrients + secondary nutrients; T4: macronutrients + secondary nutrients + micronutrients) were cross-combined with two initial AN levels (100 and 150 mg/L). The objectives were to quantify the daily removed amount and removal efficiency for AN, COD, and TP (Equations 1, 2), and to assess the coupled effects of loading rate and nutrient supplementation. AN levels were adjusted on a weekly basis to maintain the target load, and each treatment was performed in triplicate. The element addition schemes are shown in Table 1. The calculation formulas for daily removal amount and removal rate are as follows:

$$R_{area}=\frac{M}{A\times t}$$ (1)

$$RE\left(\%\right)=\frac{M}{M_{added}}\times 100\%$$ (2)

Where:

*R*_*area*_ is the daily removal amount per unit area (g·m^-2^·d^-1^);

*M* is the total mass of pollutant removed (mg); *A* is the planting area (m^2^);

*t* is the experimental duration (d);

*M*_*added*_ is the total mass of a specific nutrient (e.g., AN, TP, or COD) in the added biogas slurry (mg).

### 2.3 Measurement indicators and methods

#### 2.3.1 Plant growth and quality indicators

At harvest, plant height (tape measure), root length (ruler), and fresh/dry weight (analytical balance, 0.001 g) were recorded for five randomly selected plants per box. Chlorophyll content was determined by UV spectrophotometry; soluble sugars by anthrone colorimetry; soluble proteins by the Folin-phenol method; and reduced vitamin C by molybdenum blue colorimetry. Details of the instruments and equipment used are presented in Supplementary Table S2.

#### 2.3.2 Biogas slurry analysis

AN, TN, and COD were measured by Nessler’s reagent spectrophotometry, alkaline persulfate digestion-UV spectrophotometry, and potassium dichromate colorimetry method, respectively. pH and EC were measured by pH and conductivity meters, and elemental concentrations was determined by inductively coupled plasma optical emission spectrometry (ICP-OES). Details of the instruments and equipment used are presented in Supplementary Table S2.

### 2.4 Statistical analysis

Data were processed using Microsoft Excel 2019 and GraphPad Prism 8.0. All values are presented as means ± standard deviations, and differences were considered significant at p < 0.05.

## 3 Results and discussion

### 3.1 Regional variation in biogas slurry pollution and the EC-Based rapid detection model

Biogas slurries from Northeast China, Jiangsu, and Sichuan represent cold, environmentally regulated, and ecologically fragile regions, respectively, and exhibited significant differences in AN, TN, and COD loads that necessitate regional calibration. The measured physicochemical parameters are summarized in Table 2.

**TABLE 2:** Characteristics of biogas slurry from Northeast, Jiangsu, and Sichuan regions.

Pig farm source	EC (mS/cm)	pH	AN (mg/L)	TN (mg/L)
Northeast 1	7.42	7.89	808.75	820.10
Northeast 2	9.24	8.13	856.88	872.60
Northeast3	5.19	8.47	319.38	346.40
Northeast4	3.38	7.84	156.25	161.10
Northeast5	4.95	7.75	448.13	493.10
Northeast6	5.65	8.18	404.38	416.20
Northeast7	6.42	7.92	618.75	630.50
Northeast8	6.17	7.86	551.88	583.50
Northeast9	8.66	7.41	1,102.19	1,185.75
Jiangsu1	4.39	7.59	306.88	315.90
Jiangsu2	4.32	7.46	346.40	380.63
Jiangsu3	5.10	7.84	391.10	440.63
Jiangsu4	6.10	7.75	507.50	546.88
Jiangsu5	4.60	7.50	369.60	387.50
Jiangsu6	3.59	7.42	316.80	326.25
Jiangsu7	3.19	7.70	163.80	185.94
Sichuan1	6.60	8.06	569.20	607.50
Sichuan2	7.90	8.08	741.10	813.13
Sichuan3	4.94	7.44	455.00	470.80
Sichuan4	5.68	8.13	458.00	482.50
Sichuan5	5.22	8.37	453.80	496.88
Sichuan6	5.38	7.74	463.60	521.25
Sichuan7	7.21	8.09	667.70	713.13

Northeastern slurries exhibited the highest and most variable AN and TN concentrations, indicating the greatest treatment challenge; Jiangsu had the lowest nitrogen loads; and Sichuan was intermediate. The AN/TN ratio exceeded 88% in all samples, confirming complete anaerobic conversion of organic nitrogen to ammonium (Wilińska-Lisowska et al., 2023). EC was primarily contributed by ammonium ions and showed stable linear correlations with AN and TN, providing a basis for rapid detection. The pH range (7.41–8.47) was neutral to slightly alkaline, with minimal interference on the EC-nitrogen relationship. Therefore, local EC calibration is required for accurate load estimation.

Linear regressions between EC and AN/TN were significant (p < 0.05) for all regions, with best fits for Northeast and Sichuan (Figures 1a–f). Validation using local Sichuan samples, however, showed that the original equations underestimated actual concentrations (Figures 1g,h), likely due to continued microbial ammonium generation, temperature effects on EC, and interference from organic ions (Wilińska-Lisowska et al., 2023; Yadav et al., 2023). After coefficient correction, the deviation between predicted and measured values decreased substantially. The corrected AN model (R^2^ = 0.926) was more accurate than the TN model (R^2^ = 0.805), reflecting less interference from salts and organics.

**FIGURE 1:**
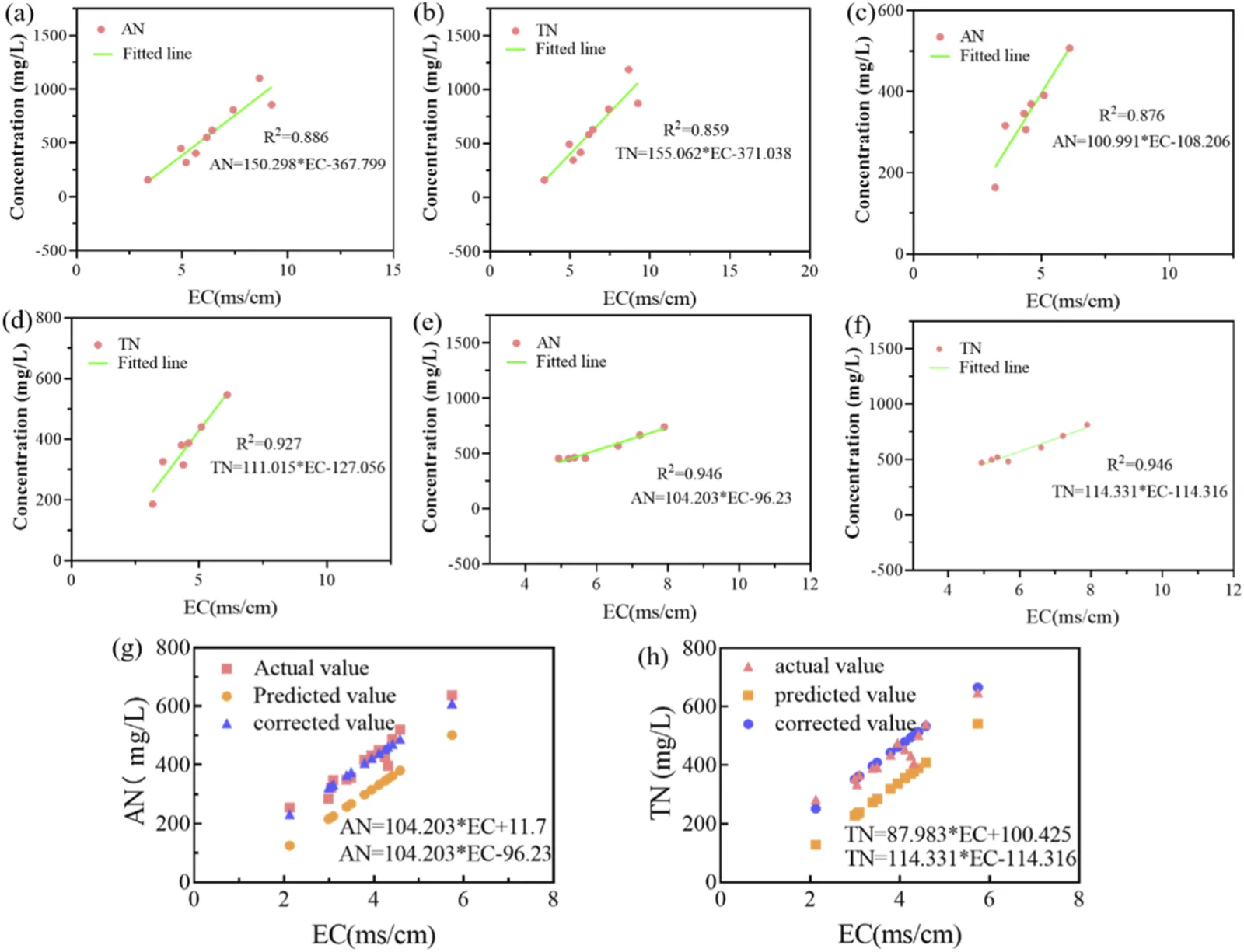
Coupled regression models between electrical conductivity and major nutrient indices (AN and TN) in biogas slurry, and the corrected results for Sichuan Province. **(a)** AN in Northeast China; **(b)** TN in Northeast China; **(c)** AN in Jiangsu Province; **(d)** TN in Jiangsu Province; **(e)** AN in Sichuan Province (original); **(f)** TN in Sichuan Province (original); **(g)** Corrected AN in Sichuan Province; **(h)** Corrected TN in Sichuan Province.

The final calibrated equations were:

$$\text{AN }\left(\text{mg}/L\right)=104.203\times \text{EC}+11.7\;\left(R^{2}=0.926\right)$$ (3)

$$\text{TN }\left(\text{mg}/L\right)=87.973\times \text{EC}+100.425\;\left(R^{2}=0.805\right)$$ (4)

These equations (Equations 3, 4) enable on-site EC measurement to precisely control incoming AN loads, preventing toxic shocks and system failure.

### 3.2 Effect of ammonium nitrogen load on the physiological activity of *Ipomoea aquatica* Forssk.

#### 3.2.1 Tolerance threshold

To determine the safe ammonia nitrogen concentration for the stable operation of the hydroponic system and the toxic threshold of *I. aquatica* inactivation, raw biogas was diluted to six ammonium levels (50, 100, 150, 200, 300, 400 mg/L). The gradient test showed that at 100 mg/L AN, plants developed thick main roots with numerous lateral branches (Figure 2b), resulting in the maximum absorptive surface area and pollutant removal potential (Mufarrege et al., 2023). With slight ammonium stress (150 mg/L), I. aquatica's growth was slightly weaker than that at the optimal group (100 mg/L), but the plants could still grow normally (Figure 2c). At 50 mg/L, plant biomass was relatively low due to insufficient nitrogen availability in the water (Figure 2a). When the ammonium level exceeded 200 mg/L, ammonium toxicity became evident, as manifested by root shrinkage, a significant reduction in plant height, and decreased physiological activity (Figure 2d). At the high pollution loads of 300 and 400 mg/L, widespread root rot and seedling death occurred, leading to the intact root system being no longer observable (Figures 2e,f).

**FIGURE 2:**
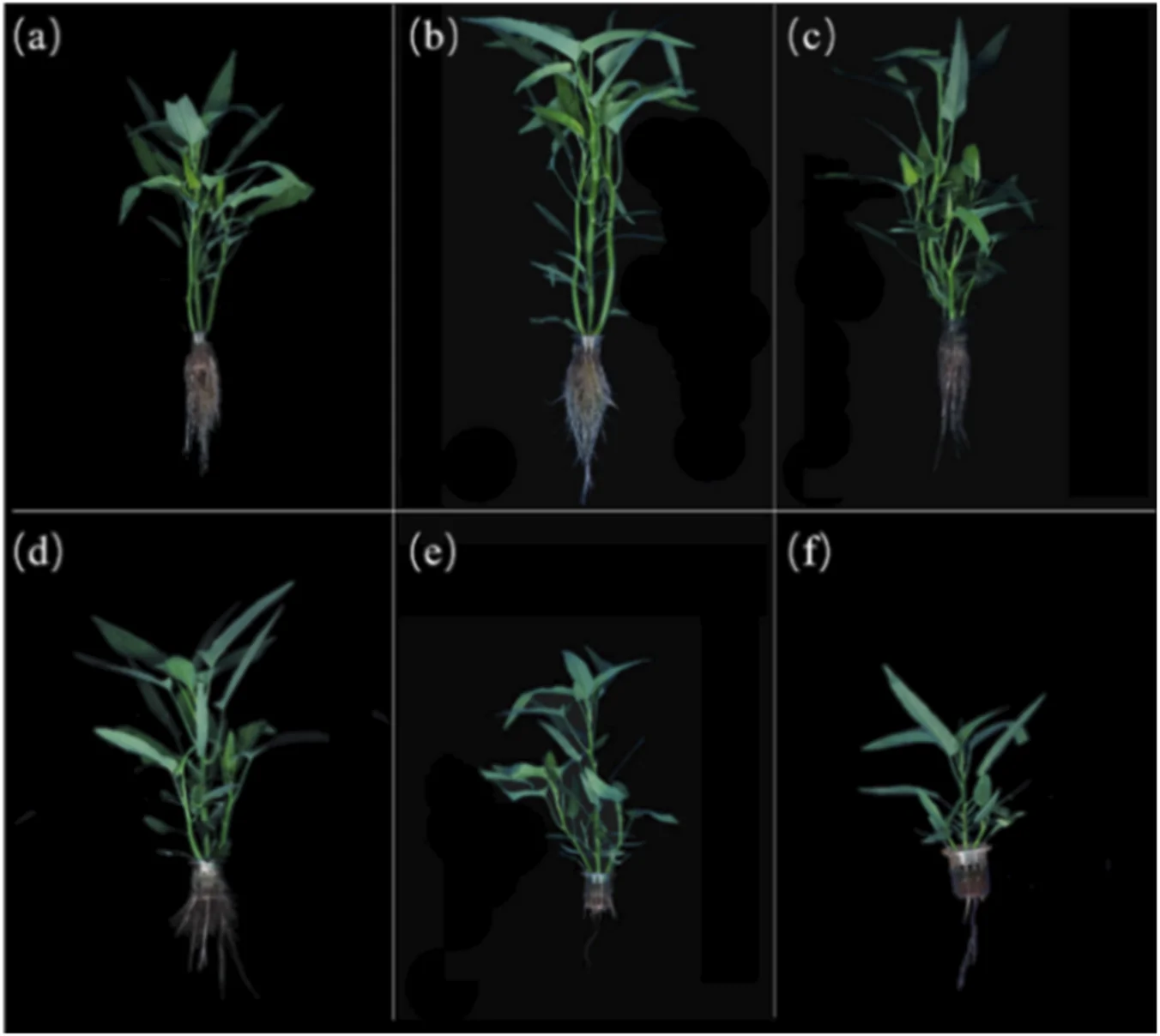
Growth performance of hydroponic *Ipomoea aquatica* at different ammonium nitrogen concentrations 7 days after transplanting: **(a)** 50 mg/L, **(b)** 100 mg/L, **(c)** 150 mg/L, **(d)** 200 mg/L, **(e)** 300 mg/L, and **(f)** 400 mg/L.

The yield of hydroponic *I. aquatica* was the highest at 100 mg/L AN, reaching 889 kg/mu (1 mu = 1/15 ha), which was significantly better than that of other treatment groups (Table 3). Thus, 100 mg/L AN is the optimal concentration for the growth and yield of hydroponic *I. aquatica*, it is in close agreement with the observations of literature (Xu J. et al., 2025), while 200 mg/L AN is the critical tolerance threshold. In actual engineering, the AN concentration of the diluted biogas slurry should be controlled within 200 mg/L, and 100 mg/L is the optimal hydroponic concentration.

**TABLE 3:** Yield of hydroponic *Ipomoea aquatica* under different ammonium nitrogen concentrations.

An concentrations (mg/L)	*I. aquatica* Yield(g)	Converted yield (kg/mu)
50	325 ± 21 b	525 b
100	550 ± 141 a	889 a
150	425 ± 17 ab	687 ab
200	400 ± 28 ab	646 ab
300	150 ± 28 c	242 c

#### 3.2.2 Effects of ammonium nitrogen concentration on photosynthetic and quality traits of *Ipomoea aquatica*

AN level significantly altered the concentrations of chlorophyll, soluble sugars, proteins, and vitamin C, indicating physiological responses to ammonium stress (Domínguez-Valdivia et al., 2008). As shown in Figures 3a–c, soluble sugar content in T3 (post-harvest increase to 150 mg/L) was significantly higher than in T1, T2, and T4, suggesting that additional ammonium after harvest promoted sugar accumulation for stress resistance. Sugars in T1, T2, and T3 were all higher than in T4, confirming an ammonium stress response, consistent with previous reports of a unimodal response of soluble sugars and proteins to nitrogen supply (Li et al., 2026). Soluble protein content followed the order T4 > T1 > T2 > T3, with the low value in T3 indicating protein degradation under high nitrogen stress. Vitamin C content increased with slurry concentration, suggesting upregulation of antioxidant synthesis under stress. The generally suboptimal quality might also be attributed to micronutrient deficiencies after dilution. Chlorophyll a content was the highest in T1, followed by T4, T3, and T2 (Figure 3d); chlorophyll b was the highest in T4. High AN concentrations reduced both chlorophyll a and b, and the prolonged high load in T3 caused a continuous decline in chlorophyll, indicating persistent photosynthetic damage (Alencar et al., 2019).

**FIGURE 3:**
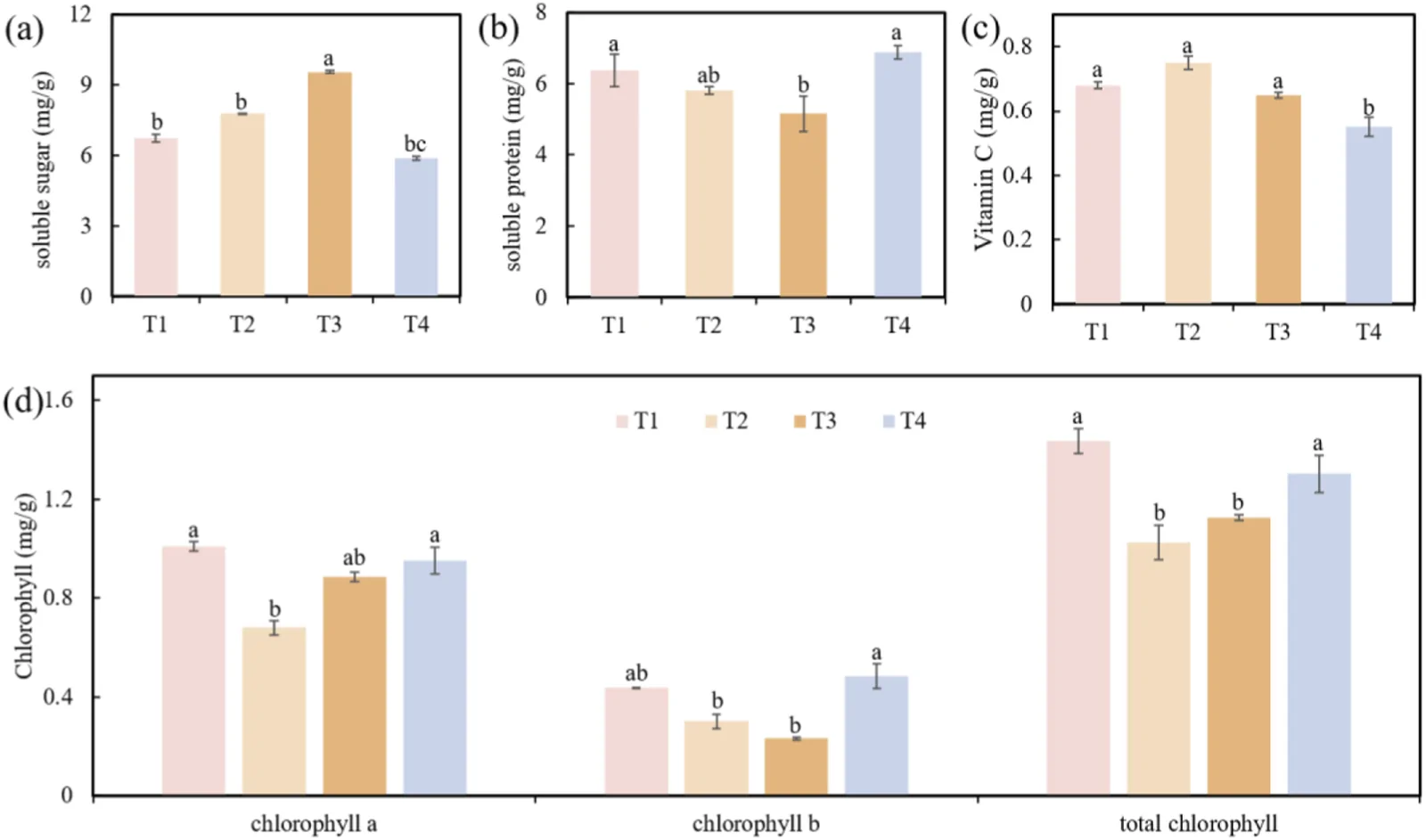
Quality indicators of *Ipomoea aquatica* under different AN concentrations: **(a)** soluble sugar; **(b)** soluble protein; **(c)** vitamin C; and **(d)** chlorophyll.

Although soluble sugars and vitamin C accumulated as stress metabolites, they could not offset the damage to roots and photosynthesis (Xu H. et al., 2025). Soluble protein degradation under high ammonium might inhibit key enzymes for nitrogen and phosphorus metabolism, potentially reducing the plant’s pollutant uptake capacity. Therefore, both growth and quality indicators confirm 100 mg/L as the optimal concentration.

### 3.3 Regulatory effects of nutrient Co-Application on *Ipomoea aquatica* activity

Dilution to safe AN levels led to severe deficiencies in Mg, Fe, Mn, B, and other micronutrients. Sole use of the diluted slurry could not sustain adequate physiological activity, thereby constraining both yield and nutrient uptake (Wang et al., 2019). As shown by plant height, root length, fresh weight, and dry weight results (Figures 4a–c), the full-element treatment (T4) outperformed all others except the CK, with the highest yield and the largest root surface area for pollutant absorption. Treatments with micronutrients significantly enhanced growth over those without, while macro-only (T2) or macronutrients and secondary nutrients (T3) treatments showed no marked improvement (Figures 4b,c), highlighting that micronutrients are essential for yield and root development.

**FIGURE 4:**
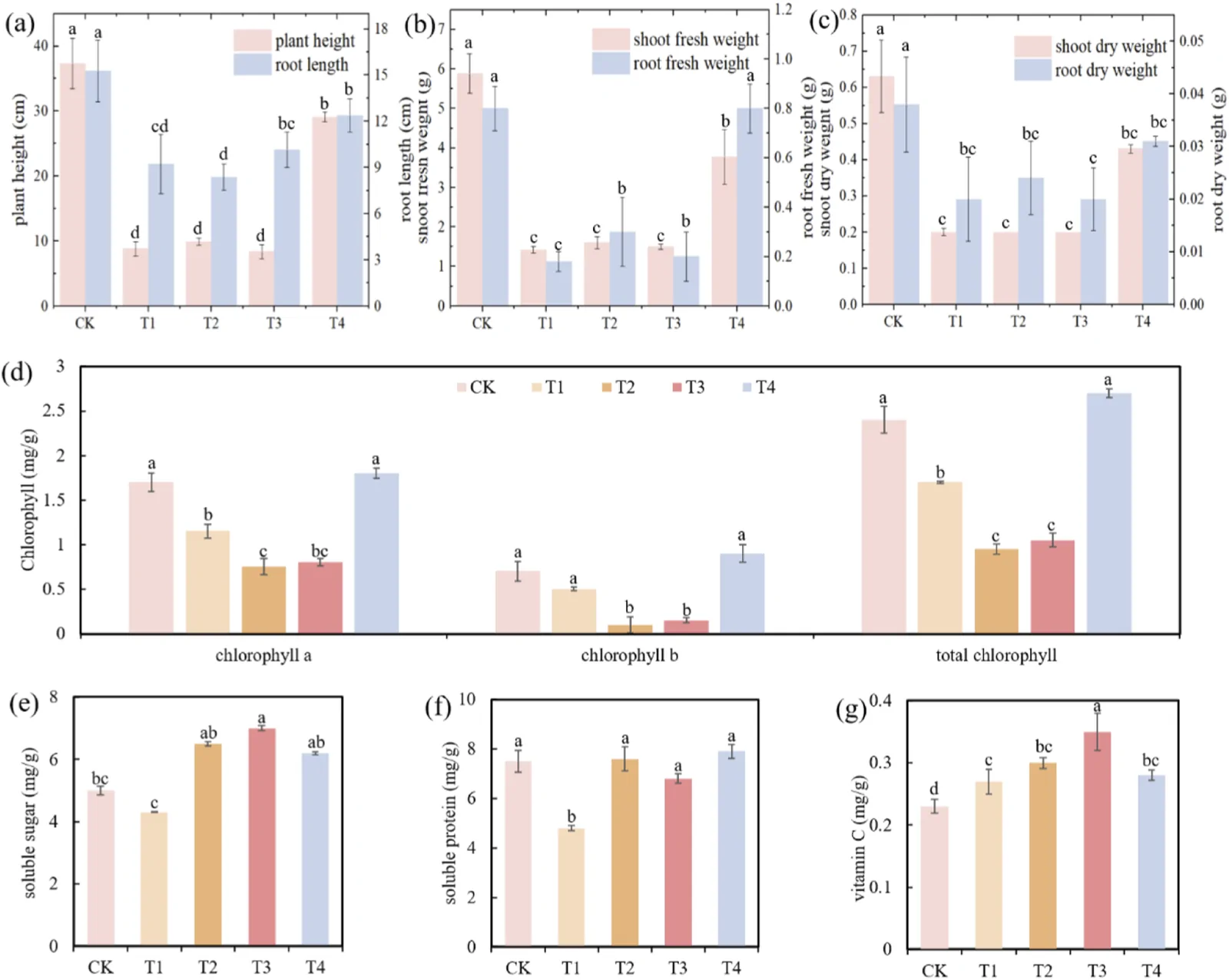
Effects of different element supplementation on the growth and quality of *I*. *aquatica*: **(a)** plant height and root length; **(b)** shoot and root fresh weight; **(c)** shoot and root dry weight; **(d)** chlorophyll; **(e)** soluble sugar; **(f)** soluble protein; and **(g)** vitamin C.

Chlorophyll content was lowest in T1 (without supplementation, Figure 4d), indicating photosynthetic impairment, while T4 was comparable to CK. All supplemented treatments showed consistently higher quality indicators than T1 (Figures 4e–g), reflecting stronger stress tolerance and sustained pollutant uptake (Yu et al., 2013). T1, fed only with diluted slurry, suffered from poor physiological condition and low N/P removal, highlighting the need for targeted nutrient addition (Wang et al., 2019). Overall, supplementing with a complete set of macronutrients, secondary nutrients, and micronutrients simultaneously enhanced yield, quality, and pollutant removal capacity.

### 3.4 Purification performance of the hydroponic system

#### 3.4.1 Ammonium nitrogen removal performance of the hydroponic system

The daily removal amounts and removal efficiencies of AN, COD, and TP are presented in Figure 5. The daily AN removal amount was significantly higher at 150 mg/L than at 100 mg/L (Figure 5a), indicating that a higher initial concentration promoted absolute uptake. Treatment T2 (macro-only) showed lower removal, likely owing to the lack of secondary nutrients and micronutrients, which restrained ammonium removal (likely due to inhibited root uptake) (Chen et al., 2015). Supplementation with secondary elements (T3) improved removal by optimizing enzyme activities and cell membrane stability. Full micronutrients (T4) resulted in a slight reduction compared with T3, possibly due to ionic antagonism or mild microbial stress. T1 (no addition) showed intermediate values. Despite the higher absolute AN removal at 150 mg/L, the removal efficiency per unit substrate was slightly lower, implying substrate inhibition (Figures 5a,b).

**FIGURE 5:**
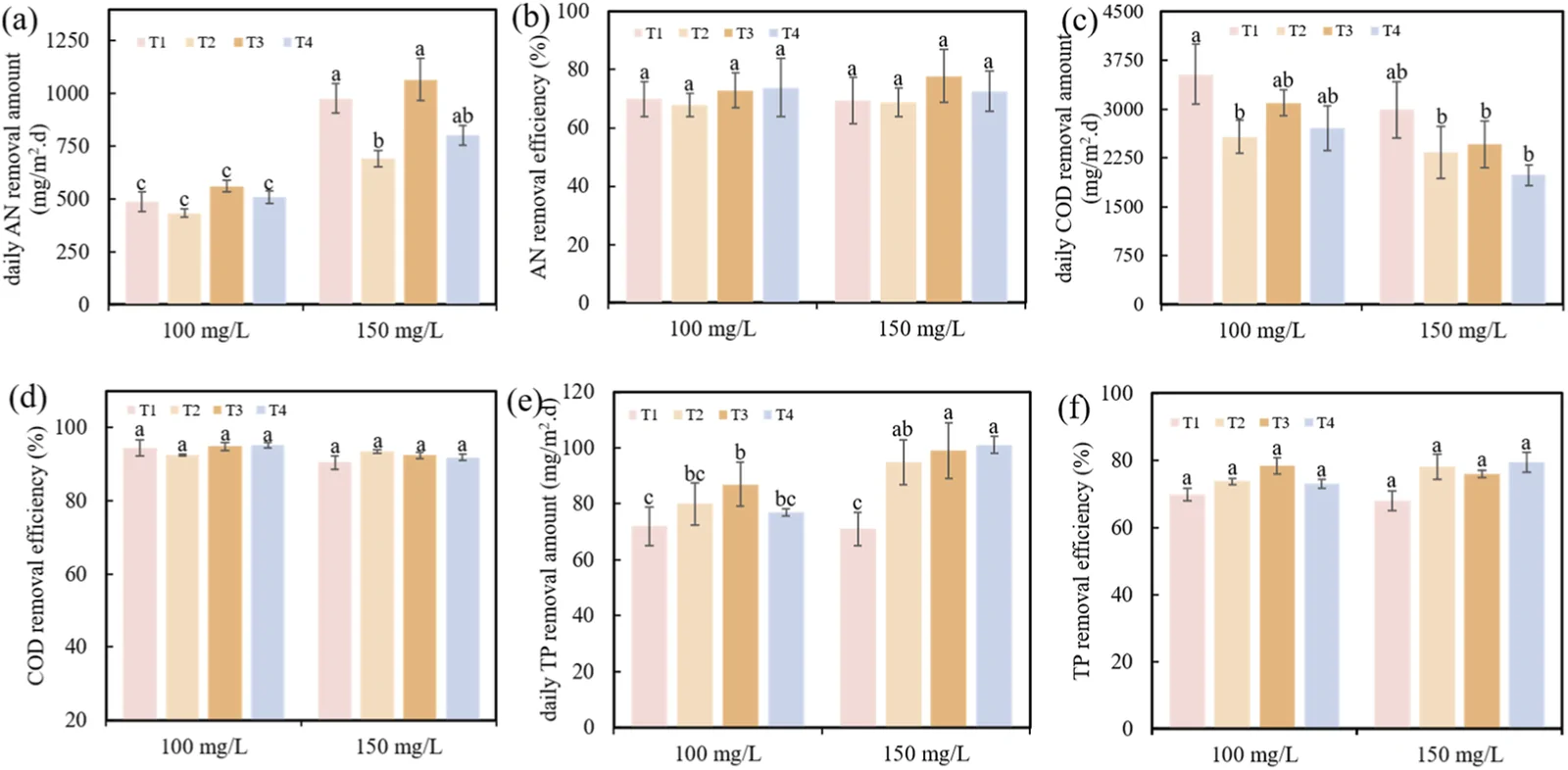
Daily removal amount and removal efficiencies of AN, COD, and TP by hydroponic *Ipomoea aquatica*: **(a)** daily AN removal amount; **(b)** AN removal efficiency; **(c)** daily COD removal amount; **(d)** COD removal efficiency; **(e)** daily TP removal amount; and **(f)** TP removal efficiency.

#### 3.4.2 COD removal performance of the hydroponic system

Xu et al. (Xu et al., 2024) proposed that rhizosphere microorganisms of *I. aquatica* are involved in pollutant removal, and that the synergistic interaction between *I. aquatica* and microorganisms is an effective approach for pollutant removal. Due to the well-developed aerenchyma in its roots (Figure 2b), *I. aquatica* can transport oxygen absorbed from the aboveground parts to the rhizosphere, creating aerobic microzones at the root–water interface. These microzones provide essential conditions for aerobic heterotrophic microorganisms to degrade organic pollutants. Simultaneously, the roots of *I. aquatica* secrete organic acids, soluble sugars, amino acids, and other substances into the rhizosphere. These root exudates serve as readily available carbon sources for microorganisms, significantly enhancing microbial abundance and metabolic activity, and thereby accelerating the co-metabolic degradation of complex organic compounds in biogas slurry. This is consistent with the findings of Lin et al. (Lin et al., 2023), who reported that *I. aquatica* exhibits a radial oxygen loss (ROL) rate of 2.1 µmol/(g·h) and a root exudate production of 8.54 mg/(g·d), providing ample electron acceptors and carbon substrates for rhizosphere microorganisms. This synergistic mechanism is the core driver for the sustained removal of COD in the system. Notably, the addition of exogenous mineral nutrients significantly reduces the total single-batch daily COD removal amount of the system (Figure 5c), and this reduction becomes more pronounced as the variety of supplemented nutrients increases. This phenomenon may be attributed to the alteration of plant organic metabolism and subsequent changes in the composition and release of root exudates induced by exogenous mineral elements (Hosseinzadeh et al., 2017). Nevertheless, neither the initial ammonium nitrogen concentration nor the supply of macro- and micro-nutrients significantly affected the final COD removal efficiency. All treatment groups consistently achieved COD removal efficiencies above 90% (Figure 5d), demonstrating that the hydroponic *I. aquatica* system is effective in removing COD from biogas slurry.

#### 3.4.3 Phosphorus removal performance of the hydroponic system

The TP removal results (Figures 5e,f) indicated that nutrient addition significantly enhanced TP removal; however, the optimal nutrient formulation exhibited a clear dependence on AN concentration. Under a low AN (100 mg/L) condition, the order of TP removal efficiency was T3 > T2 > T4 > T1. In particular, the T4 treatment, which received the full complement of micronutrients, exhibited lower efficiency than T3 (which lacked micronutrients). This may be attributed to the enhanced selectivity of root plasma membrane cations under nitrogen-limited conditions, where the additionally supplied micronutrient cations (especially Fe^2+^ and Zn^2+^) compete with H_2_PO_4_^−^ for root surface binding sites and the proton co-transport channels of phosphate transporters (such as the PHT1 family), thereby inducing competitive inhibition of ion channels and reducing the inward flux of phosphorus (Chen et al., 2015). On the other hand, the introduction of micronutrients may selectively inhibit the activity of certain phosphate-solubilizing bacteria (PSB), leading to a decline in the remineralization rate of immobilized phosphorus, which is a phenomenon we refer to as “mild microbial stress” (Hosseinzadeh et al., 2017).

When the AN concentration was elevated to 150 mg/L, the order completely reversed to T4 > T3 > T2 > T1, indicating that high nitrogen supply modulated the inter-element relationships. On the one hand, under high nitrogen conditions, the accelerated plant growth rate and enhanced root metabolic activity increased the demand for ATP and reducing power, inducing greater expression of phosphate transporters. Consequently, the additional uptake sites were no longer saturated, and the competitive effects of micronutrients were diluted (Chen et al., 2026). On the other hand, high nitrogen removal required higher activities of key enzyme systems, such as Zn-dependent alkaline phosphatase (ALP) and Fe-dependent peroxidase (POD) (Browning et al., 2017; Saavedra et al., 2025), the activities of which became secondary bottlenecks for phosphorus removal under high nitrogen conditions. The supplementation of micronutrients precisely alleviated this limitation.

In summary, under low AN conditions, micronutrients and phosphate exhibited an antagonistic relationship, whereas under high AN conditions, they transitioned to a synergistic relationship. This antagonism–synergism shift reveals the underlying mechanism by which initial nitrogen loading indirectly regulates phosphorus removal efficiency through alterations in root uptake kinetics and the elemental interaction network. These findings suggest that under low loading conditions, priority should be given to ensuring the supply of secondary nutrients, while under high loading conditions, precise supplementation of the full suite of micronutrients should be implemented.

### 3.5 Economic evaluation

#### 3.5.1 Calculation of the net value of biogas slurry

The optimal operational protocol integrated dilution of raw biogas slurry to 100 mg/L ammonium nitrogen with full macronutrients, secondary nutrients, and micronutrients, and adjusting the load on a weekly basis (Figure 6a). With a pond depth of 15 cm, the hydroponic system contains 99 m^3^ of working solution per mu. The system can assimilate 57.4 m^3^ of high-pollution slurry per mu per year (Figure 6b), achieving harmless reduction of AN, COD, and TP, and eliminating the costs of chemicals, energy, and sludge disposal in conventional treatments.

**FIGURE 6:**
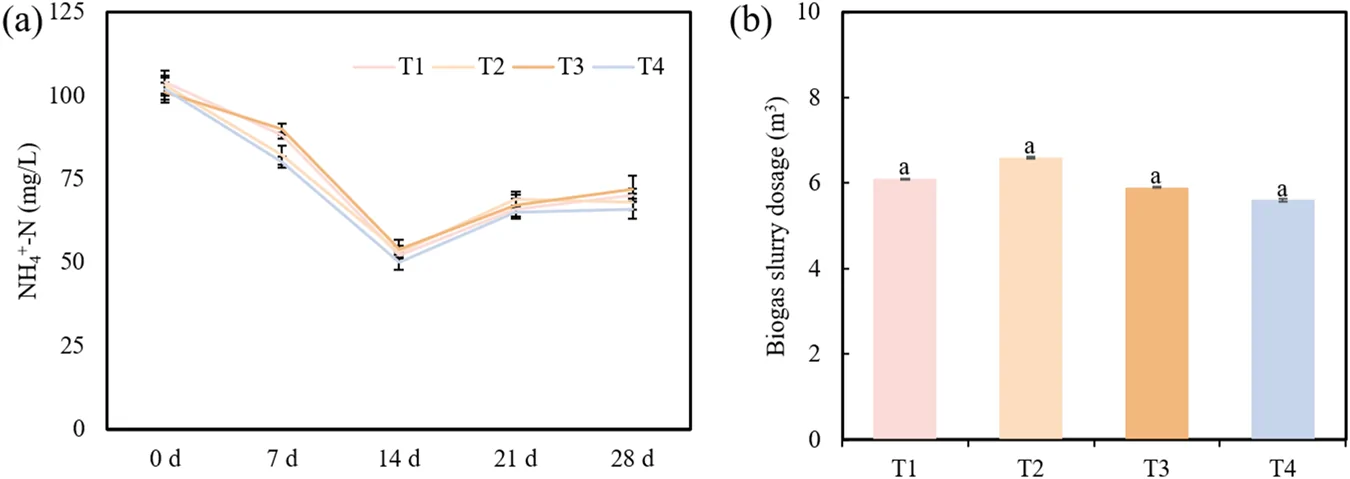
**(a)** Changes in ammonium nitrogen concentration, and **(b)** biogas slurry input in hydroponic systems across different treatment groups.

Based on the nutrient formulation, the amount of commercial nutrient solution replaced by slurry was calculated. The formulation requires additional P, K, Ca, Mg, S, Fe, Cu, Zn, Mn, Mo, and B. Prices of supplements are listed in Table 4. Market investigation showed that the cost of a full commercial nutrient solution is approximately CNY 2200 per mu per batch. During the cultivation cycle (April–October; first crop 30 days, subsequent cuttings 20 days, 4–5 harvests per batch, two batches per year), about 28.7 tons of slurry is used per mu per batch. The supplemental nutrient cost is approximately CNY 262.7. Thus, 28.7 tons of slurry replaces CNY 1937.3 worth of commercial nutrients. After deducting transport costs (CNY 10 per ton), the net value of the slurry is approximately CNY 57.5 per ton.

**TABLE 4:** Quantity and price of supplementary nutrients.

Supplementary fertilizer name	Application rate (kg/mu)	Unit price (CNY/kg)	Cost (CNY/mu)
KH_2_PO_4_	6.69	10.00	66.90
KCl	5.88	3.60	21.20
MgSO_4_·7H_2_O	19.62	5.00	98.10
CaCl_2_	9.94	6.20	61.60
Micronutrient fertilizer	30–50 mL/mu	50.00 CNY/L	15.00
Total	​	​	262.7

#### 3.5.2 Net income of the hydroponic system

##### 3.5.2.1 Initial investment

The initial investment costs of the hydroponic system are shown in Table 5. Given that this study aims to provide a reference for the treatment of biogas slurry from small- and medium-scale pig farms in China, the hydroponic ponds need to be constructed on-site, thus incurring corresponding civil engineering costs. The total initial investment of the system is CNY 63,898, with an average annual amortization cost of CNY 16,337.

**TABLE 5:** Initial investment of the hydroponic system.

Item	Description	Unit	Unit value (CNY)	Quantity	Initial cost (CNY)	Useful life year (year)	Annual cost (CNY)
Hydroponic pond construction	Concrete construction	mu	20,000	1	20,000	15	1,333
Anti-seepage membrane	Full coverage laying of the hydroponic pond	m^2^	20	667	13,340	2	6,670
Floating raft	560 mm (L) × 380 mm (W)×30 mm (T)	Piece	10	2,778	27,780	5	5,556
Planting baskets	12 planting baskets per floating raft set	set	1	2,778	2,778	1	2,778
Subtotal	—	—	—	—	63,898	—	16,337

##### 3.5.2.2 Operating cost

The annual operating costs of the hydroponic system are detailed in Table 6, totaling CNY 51,030. Among these, because the biogas slurry can supplement part of the nutrient elements, the average annual fertilizer expenditure is only CNY 530, accounting for a very low proportion of the total operating costs. In contrast, *I. aquatica* is cultivated twice a year, with 4–5 harvests per crop, resulting in labor costs as high as CNY 40,000, which account for 78.4% of the total operating costs.

**TABLE 6:** Operating costs of the hydroponic production system.

Item	Description	Unit	Unit value (CNY)	Quantity	Total value (CNY)
Seedlings	Willow-leaf cultivar	seedling	0.15	60,000	9,000
Land rental fee	Prices may vary by location, ranging from 500–CNY 2,000 per mu	mu	1,500	1	1,500
Labor cost	Includes planting and harvest	—	40,000	1	40,000
Fertilizer	Nutrient composition varies among batches of biogas slurry; average price used	Batch	263	2	530
Subtotal	—	—	—	—	51,030

##### 3.5.2.3 Economic evaluation

This economic evaluation is based on the premise that all vegetable quality indicators meet food safety standards (detailed testing indicators are provided in the SI Text S5). The annual yield of hydroponic *I. aquatica* is approximately 45,000 kg/mu, with a market price of about CNY 2.5 per kg, giving total annual income of CNY 112,500 per mu. After deducting the annual amortization and operating costs, the net profit per mu is CNY 45,133.

### 3.6 Research limitations and future perspectives

#### 3.6.1 Application boundaries of the EC model and improvement directions

The EC-AN regression model developed in this study was calibrated using raw biogas slurry samples, in which ammonium was the dominant ionic species. The model performed well for estimating initial AN loads prior to the addition of exogenous mineral salts. However, EC reflects bulk solution ionic strength rather than serving as a selective indicator of ammonium concentration. In treatments supplemented with exogenous nutrients, the added K^+^, Ca^2+^, and Mg^2+^ salts contributed substantially to the EC signal, making it impossible to distinguish ammonium-derived EC from non-ammonium-derived EC contributions using linear regression alone. Furthermore, selective ion uptake by plant roots and water evaporation continuously altered the ionic composition, rendering the static linear model inadequate for accurately capturing real-time AN variation in the dynamic system. Therefore, this model is not applicable for real-time AN tracking in treatments receiving synthetic nutrient supplementation. Considering the practical needs of small- and medium-scale pig farms in China, we recommend adopting portable ammonia nitrogen detection devices as a complementary or alternative approach to the EC model, to effectively address the routine monitoring of AN concentrations in biogas slurry hydroponic systems. The inherent limitation of the EC model in supplemented treatments is a methodological constraint that future studies must acknowledge and address.

#### 3.6.2 Toward mechanistic understanding of pollutant removal

In this study, COD removal efficiency consistently exceeded 90% across all treatments and were not significantly affected by AN load or nutrient addition, suggesting that COD removal was primarily driven by rhizosphere microbial processes rather than direct plant uptake. The root system of *I. aquatica* creates aerobic microzones at the root-water interface through radial oxygen loss (ROL), providing electron acceptors for aerobic degrading microorganisms. Meanwhile, root exudates act as “priming carbon sources” that stimulate microbial metabolic activity and accelerate the co-metabolic degradation of organic matter (Liang et al., 2023). However, as we did not directly characterize the rhizosphere microbial community structure and function, the above mechanisms remain indirect inferences. Regarding nutrient mass balance, due to experimental constraints, gaseous nitrogen losses and phosphorus precipitate forms were not directly measured. Future studies should integrate metagenomics with functional gene quantification to establish causal links between microbial composition, function, and treatment efficiency. Stable isotope probing (SIP) techniques should be employed to trace organic carbon flow through the plant-microbe system, and dynamic closed-chamber methods coupled with gas chromatography should be introduced to precisely quantify gaseous nitrogen losses. These efforts will enable the construction of a complete mass balance framework, providing reliable data support for system optimization and the development of hydroponic purification systems with higher resource recovery efficiency.

#### 3.6.3 Temporal and spatial scale constraints

This study was conducted over a single growing season (April–October) at a single location (Chengdu, Sichuan Province), and thus has the following spatial and temporal limitations: under winter low-temperature conditions (<15 °C), the growth of *I. aquatica* ceases and microbial activity declines, potentially leading to a substantial decrease in system purification capacity; the applicability of this system to different climatic zones (e.g., cold temperate regions of Northeast China, tropical/subtropical regions of South China) and different biogas slurry sources (e.g., pig manure, cattle manure, food waste digestate) has not been verified; and regional variations in fertilizer prices, labor costs, and transportation expenses may significantly affect economic feasibility. Therefore, we recommend that future studies conduct localized hydroponic system trials across multiple representative climatic zones and livestock production systems, to validate the stability and adaptability of this technology under diverse scenarios, thereby providing more robust technical support for the nationwide resource utilization of biogas slurry.

## 4 Conclusion

1. Rapid monitoring tool: EC-based linear regression models (AN = 104.203 × EC + 11.7, R^2^ = 0.926; TN = 87.973 × EC + 100.425, R^2^ = 0.805) enable rapid initial estimation of AN and TN loads in raw biogas slurry, supporting on-site load adjustment. However, these models are not suitable for real-time tracking after exogenous nutrient addition due to interference from other ions.
2. Optimal ammonium loading: The safe and productive AN concentration for *I. aquatica* hydroponics is 100 mg/L, yielding the highest biomass (889 kg/mu) and robust root development; 200 mg/L is the critical toxicity threshold beyond which root damage and mortality occur.
3. Nutrient supplementation strategy: Dilution to 100 mg/L AN causes micronutrient deficiencies. Full supplementation with macro-, secondary, and micronutrients (T4) significantly enhances plant growth, chlorophyll content, soluble sugars, proteins, and vitamin C, outperforming partial supplementation or no addition.
4. Purification performance: The system consistently removes >90% of COD across all treatments, driven by rhizosphere microbial activity supported by root oxygen loss and exudates. TP removal is regulated by AN load: under low AN (100 mg/L), secondary nutrients (T3) are most effective, while under high AN (150 mg/L), full micronutrient supplementation (T4) is required, revealing a load-dependent antagonism-synergism shift between micronutrients and phosphate uptake.
5. Economic viability: With an annual yield of 45,000 kg/mu and the net profit per mu is CNY 45,133.

These findings offer a scientific basis and operational framework for the resource-oriented utilization of biogas slurry, contributing to circular agriculture and livestock pollution control. Future work should focus on elucidating microbial mechanisms, establishing complete mass balances, and validating system performance across diverse climatic and operational conditions.

## Data Availability

The raw data supporting the conclusions of this article will be made available by the authors, without undue reservation.
