# Supplementary material for: Coupling relationships between yield traits of Ipomoea aquatica Forssk. and biogas slurry purification under varied nutrient supplementation regimes
Source: Front Bioeng Biotechnol. 2026 Oct 1;14:1927455. doi: 10.3389/fbioe.2026.1927455 (PMC13668878; doi:10.3389/fbioe.2026.1927455)
Supplement: Supplementary file 1 [file Supplementaryfile1.docx]

Supporting Information

Coupling Relationships Between Yield Traits of *Ipomoea aquatica* Forssk. and Biogas Slurry Purification Under Varied Nutrient Supplementation Regimes

Yuhuan Wu^1,2,3^, Lanmei Liang^2,3^, Linyu Xie^2,3^, Xujing Guo^1^, Wenguo Wang^2,3^, Liangwei Deng^2,3^, Lan Wang^2,3^, Hongwei Zhang^2,3^, Ting He^2,3^, Peike Wu^2,3^, Yunhong Zhang^2,3*^

^1^College of Resources and Environment, Chengdu University of Information Technology, No. 24 Block 1, Xuefu Road, Chengdu, 610225, PR China

^2^Biogas Institute of Ministry of Agriculture and Rural Affairs, No. 13, Section 4, Renmin South Road, Chengdu 610041, PR China

^3^Key Laboratory of Development and Application of Rural Renewable Energy, Ministry of Agriculture and Rural Affairs, Chengdu, 610041, PR China

* Correspondence:

Yunhong Zhang

zhangyunhong@caas.cn

**Text S1: Information on pig farms**

Northeast China: Suihua in Heilongjiang Province has a cold temperate continental monsoon climate, with an annual mean temperature of approximately 3.4°C, annual precipitation of 511 mm, and extreme winter lows reaching as low as −42.3°C; the frost‑free period lasts 120–140 days. Siping in Jilin Province has a mid‑temperate semi‑humid monsoon climate, with a mean annual temperature of 5.7°C, precipitation of 567 mm, a frost‑free period of about 130 days, and long, cold winters. Tieling in Liaoning Province also lies in a mid‑temperate semi‑humid zone, with annual temperatures of 5–7.3°C, precipitation of 650–700 mm, and extreme minima below −31°C. Chaoyang in Liaoning Province has a northern temperate continental monsoon climate, with annual mean temperatures of 5.4–8.7°C, relatively low precipitation (450–580 mm), and a dry, windy spring prone to drought.

Jiangsu Province: Huai’an in Jiangsu Province is located in a transitional zone between the warm temperate and northern subtropical belts. It has an annual mean temperature of 13.6–14.7°C, precipitation of about 940–1000 mm, and a frost‑free period of 240 days.

Sichuan Province: Nanchong, Dazhou, Dayi, and Anyue all have a subtropical humid monsoon climate. In Nanchong, the annual mean temperature is about 17–17.5°C, with precipitation of 980–1150 mm; winters are warm, summers hot, and autumns are characterized by persistent drizzle. Dazhou has similar conditions, with annual temperatures of 16–17°C and precipitation of 1000–1200 mm. Dayi has annual temperatures of 16.1–16.7°C, precipitation of 1064–1095 mm, and a frost‑free period of 284 days. Anyue has annual temperatures of 17.4–17.6°C, precipitation of about 1026 mm, and a winter minimum of only −1.4°C, making winters mild.

Overall, Northeast China is characterized by low temperatures, low precipitation, and a short frost‑free period, whereas the Sichuan Basin has high temperatures, abundant rainfall, and a long frost‑free period. The climatic differences between the two regions are substantial, and the experimental conditions used for the EC model covered a wide range of types, from severe cold to warm and humid environments.

**Text S2: Cultivation of *I. aquatica***

Before sowing, *I. aquatica* seeds were soaked in 60°C warm water with stirring for ten minutes to break dormancy. The seeds were then germinated in a mixed soil substrate with a soil‑to‑substrate ratio of 2:1. When the seedlings reached a height of approximately 6–8 cm and had fully expanded four true leaves, they were transplanted. Prior to transplanting, the seedling soil was moistened in advance, and the seedlings were carefully uprooted to avoid damaging the roots. After removal, the roots were rinsed with clean water to remove soil and other impurities.

Ammonia nitrogen tolerance test: This experiment was conducted in an illuminated incubator. The experiment was performed in an LED light growth chamber (GZD‑1000C) purchased from Nanjing Lisigao Instrument Equipment Co., Ltd. The growth chamber was set to the following conditions: daytime duration 12 h, temperature 25°C, light intensity 12,000 Lux; nighttime duration 12 h, temperature 20°C, light intensity 0 Lux. During the hydroponic period, the humidity inside the chamber was maintained at approximately 85%.

Nutrient supplementation experiment and Pollutant Assimilation Tests: This experiment was conducted at the experimental base of Biogas Institute of Ministry of Agriculture and Rural Affairs. The base is located in a mid‑subtropical humid monsoon climate zone, characterized by mild temperatures, abundant rainfall, simultaneous warmth and humidity, synchronized water and heat, and distinct seasons. Spring temperatures rise early but are unstable; summers are hot with frequent heavy rain; autumns cool down rapidly with persistent drizzle; winters are dry and foggy. In most years, the maximum temperature reaches 33–35°C, while the minimum drops to about −3°C.

**Text S3: Preparation of the hydroponic solution**

For the biogas slurry treatment groups, macro‑ and secondary nutrients were supplemented according to a 1/2‑strength Yamazaki nutrient solution (calcium nitrate tetrahydrate 1.5 g/50 L, potassium nitrate 0.143 g/50 L, ammonium dihydrogen phosphate 0.011 g/50 L, magnesium sulfate heptahydrate 0.004 g/50 L). Micronutrients were supplemented following a general micronutrient formula (ferric sodium EDTA 1.5 g/50 L, boric acid 0.143 g/50 L, manganese sulfate 0.011 g/50 L, copper sulfate 0.004 g/50 L, zinc sulfate 0.011 g/50 L, ammonium molybdate 0.001 g/50 L), and were added directly using a commercial micronutrient fertilizer. For all element supplementation, the compounds from the Yamazaki nutrient solution were used preferentially, followed by chlorides. Because the biogas slurry came from different batches and had been stored for varying lengths of time, the basic physicochemical properties of the slurry used in the experiments differed slightly; therefore, the formulation of the hydroponic solution was adjusted for each experiment. For the specific chemical elements and fertilizers added, please refer to Table S1.

Table S1 Experimental reagents

| Chemical reagent | Reagent grade | | Manufacturer |
| --- | --- | --- | --- |
| MgSO_4_·7H_2_O | AR | | Shanghai Macklin Biochemical Technology Co., Ltd. |
| KH_2_PO_4_ | AR | | Shanghai Macklin Biochemical Technology Co., Ltd. |
| KCl | AR | | Shanghai Macklin Biochemical Technology Co., Ltd. |
| CaCl_2_ | AR | Shanghai Macklin Biochemical Technology Co., Ltd. | |
| Micronutrient fertilizer | — | Zhengzhou Zhongke Chemical Products Co., Ltd. (obtained from the local market) | |

**Text S4：Instruments and equipment**

Detailed information on instruments and equipment is provided in Table S2.

Table S2 Experimental apparatus

| Instrument name | Model | Manufacturer |
| --- | --- | --- |
| UV spectrophotometer | INESA L5S | Shanghai INESA Scientific Instrument Co., Ltd. |
| Ultrasonic cleaner | KQ‑400E | Kunshan Ultrasonic Instruments Co., Ltd. |
| COD rapid analyzer | LH‑3C | Development Co., Ltd. |
| pH meter | PHS‑3E | Shanghai INESA Scientific Instrument Co., Ltd. |
| EC meter | DDSJ‑308A | Shanghai INESA Scientific Instrument Co., Ltd. |
| Milligram balance (0.001 g) | ME103/02 | Mettler Toledo Technologies (China) Co., Ltd. |
| Inductively Coupled Plasma Optical Emission Spectrometer (ICP‑OES) | PlasmaQuant PQ9000 | Shanghai INESA Scientific Instrument Co., Ltd. |

**Note:** The electrical conductivity (EC) of the biogas slurry was measured using a DDSJ‑308A conductivity meter (Shanghai INESA Scientific Instrument Co., Ltd.). This instrument is equipped with automatic temperature compensation (ATC) function, with a temperature compensation range of 0.0–50.0 °C and an adjustable temperature compensation coefficient. During the measurement of all samples, the instrument automatically corrected the conductivity readings to a reference temperature of 25 °C to ensure comparability of results obtained under different temperature conditions. Prior to measurement, the instrument was calibrated with standard solutions, and the sample temperature was recorded for each measurement.

**Text S5: Determination of food safety parameters**

Since the water spinach grew best in the treatment group with an ammonium nitrogen concentration of 100 mg/L and full nutrient supplementation among all treatments, water spinach from this group was selected for food safety analysis. The maximum permissible limits for heavy metals and nitrite were based on the Chinese National Food Safety Standard (GB 2762‑2022), and the maximum permissible limits for microbiological indicators were based on the Chinese National Food Safety Standard (GB 29921‑2021). According to the results of heavy metals, nitrite, and microbiological indicators in water spinach (Table S3), the concentrations of all hazardous substances were within the limits stipulated by the corresponding national standards.

Table S3 The table of food safety table of liquid digestate hydroponic water spinach

| Category | Specific indicator | T4 group | Maximum permissible limit |
| --- | --- | --- | --- |
| Heavy metals (mg/kg) | Cr | Not detected | 0.5 |
|  | As | Not detected | 0.5 |
|  | Cd | Not detected | 0.2 |
|  | Hg | Not detected | 0.01 |
|  | Pb | Not detected | 0.1 |
| Nitrite (mg/kg) | Nitrite | Not detected | 20 |
| Microbiological indicators (cfu/g) | *Salmonella* | Not detected | 0 |
|  | *Shigella* | Not detected | 0 |
|  | *Staphylococcus aureus* | Not detected | 1000 |

Note：“Not detected” indicates that the concentration was below the method detection limit.
